# A High Phosphorus Diet Impairs Testicular Function and Spermatogenesis in Male Mice with Chronic Kidney Disease

**DOI:** 10.3390/nu12092624

**Published:** 2020-08-28

**Authors:** Chih-Wei Tsao, Yu-Juei Hsu, Ting-Chia Chang, Sheng-Tang Wu, Tai-Lung Cha, Chin-Yu Liu

**Affiliations:** 1 Division of Urology, Department of Surgery, Tri-Service General Hospital, National Defense Medical Center, Taipei 114, Taiwan; weisurger@gmail.com (C.-W.T.); doc20283@gmail.com (S.-T.W.); tlcha@ndmctsgh.edu.tw (T.-L.C.); 2 Division of Nephrology, Department of Medicine, Tri-Service General Hospital, National Defense Medical Center, Taipei 114, Taiwan; yujuei@mail2000.com.tw; 3 Department of Nutritional Science, Fu Jen Catholic University, Taipei 242, Taiwan; ctc5628@gmail.com

**Keywords:** chronic kidney disease, high phosphorus diet, spermatogenesis, oxidative stress, apoptosis

## Abstract

Hyperphosphatemia is a serious complication in chronic kidney disease (CKD) that occurs due to insufficient excretion of phosphorus during failure of renal function. Both CKD and an excessive phosphorus intake have been reported to increase oxidative stress and result in poor male fertility, but little is known about the reproductive function of the CKD under a poorly controlled phosphate intake. Eight-week-old C57BL/6 mice (*n* = 66) were randomly divided into four groups: a sham operation group received a chow diet as control (SC group, *n* = 14), CKD-induced mice received a chow diet (CKDC group, *n* = 16), control mice received a high phosphorus (HP) diet (SP group, *n* = 16), and CKD-induced mice received a HP diet (CKDP group, *n* = 20). CKD was induced by performing a 5/6 nephrectomy. The chow diet contained 0.6% phosphorus, while the HP diet contained 2% phosphorus. Impaired testicular function and semen quality found in the CKD model may result from increased oxidative stress, causing apoptosis and inflammation. The HP diet aggravated the negative effects of testicular damage in the CKD-induced mice.

## 1. Introduction

The rising prevalence of chronic kidney disease (CKD) has become a global serious health problem, especially in Taiwan, in which the estimated prevalence of total CKD and CKD stages 3–5 were 15.5% and 9.1%, respectively, according to a cohort study in 2018 [1], similar to the reported global prevalence (13.4% and 10.6%) [2]. Furthermore, Taiwan has had the highest incidence of end-stage renal disease (ESRD) in the world since 2000 [3,4]. With failure of renal function, numerous complications emerge, including hyperphosphatemia. An adequate phosphorus intake is essential for physiological function, as the formation of phosphorus compounds is involved in cell membrane integrity, ATP synthesis, cell signaling, skeletal growth, and mineralization [5]. Phosphate homeostasis is regulated by intestine absorption, bone, renal reabsorption, and excretion. Hormones such as parathyroid hormone (PTH), calcitriol, and fibroblast growth factor-23 (FGF-23) also play important roles in the phosphorus balance [6,7]. When the kidney function decreases to an extent, urinary excretion of phosphate lowers, and the phosphorus balance is impaired, leading to hyperphosphatemia [8].

Several studies have shown that a high serum phosphate level contributes to cardiac calcification, bone diseases, and the risk of death in CKD patients [9,10,11]. In addition, the phosphorus level was found to be positively correlated with the severity of erectile dysfunction, the inability to get or maintain an erection [12]. Low testosterone production and abnormal sperm function in CKD [13,14] or excess phosphorus [15,16] have also been observed. Furthermore, CKD was found to be linked to increasing oxidative stress, apoptosis and inflammation [17,18,19]. Moreover, phosphorus overload may induce oxidative stress and apoptosis [20], as well as inflammation [21], and these factors are involved in male infertility. Testosterone is the main sexual hormone associated with the male reproductive function, including libido activation, development of sexual organs and spermatogenesis. The production of testosterone is primarily in the Leydig cells of the testes through cytochrome P450 (CYP) and hydroxysteroid dehydrogenase (HSD) enzymes. Testosterone synthesis is also regulated by hormones secreted from the hypothalamus and pituitary gland [22]. Besides its effect on fertility, men with low testosterone concentrations may have problems in the brain, bone, and cardiovascular functions [23].

Few studies have examined male reproductive dysfunction induced by CKD with an excessive dietary phosphorus intake. Therefore, this study was designed to determine the effects of CKD and a high phosphorus (HP) diet on testosterone concentration, semen quality and spermatogenesis in male mice.

## 2. Materials and Methods

### 2.1. Animal Model and Experimental Design

Male C57BL/6 mice (*n* = 66) at 8-weeks-old were purchased from the National Laboratory Animal Center and housed in the laboratory animal center of National Defense Medical Center (Taipei City, Taiwan) after obtaining the approval from the Institutional Animal Care and Use Committee (IACUC; ethical code number: IACUC-20-186). The animals were maintained in cages under a 12 h light/dark cycle at a room temperature of 23 ± 3 °C, a 30% to 37% relative humidity and a ventilation rate of 10–15 air changes per hour. After one week of adaptation, the mice were randomly divided into two groups: control group (*n* = 30) and CKD group (*n* = 36).

The CKD mouse model was developed by performing a 5/6 nephrectomy, a two-step surgery in which two-thirds of the left kidney were removed in the first step (at 9-weeks-old), while the right kidney was completely removed after one week (at 10-weeks-old), and mice in the control group were anesthetized and submitted to laparotomy but kidneys were left intact. After surgery and observation for 3 days, both the control and CKD groups were fed a chow diet containing 0.6% phosphorus (LabDiet 5010) and a HP diet (TestDiet 1817613-203) containing 2% phosphorus for 20 weeks. The ingredients of each diet are shown in Table 1.

Briefly, this study included four groups: sham+chow diet group (SC, *n* = 14), mice received sham operation fed a chow diet; CKD+chow diet group (CKDC, *n* = 16), mice that underwent 5/6 nephrectomy fed with a chow diet; sham+HP diet group (SP, *n* = 16), mice received sham operation fed a HP diet; and CKD+HP diet group (CKDP, *n* = 20), mice that underwent a 5/6 nephrectomy fed with a HP diet. At the end of the 20-week experimental period, the mice were anesthetized with 1.2% Avertin solution, weighed and sacrificed. Blood samples were collected and centrifuged, and serum was isolated and stored at –80 °C until analysis. The reproductive system organs were weighed, and the testes were collected: some testis tissues were fixed in 10% formalin (diluted from 37% formaldehyde solution, J.T. Baker, NJ, USA) for histological evaluation, and the remainder were frozen in liquid nitrogen immediately and then kept at –80 °C for subsequent analysis. A 1-mL syringe with a 26G needle containing 0.5 mL 1× phosphate-buffered saline (PBS; diluted from 10× PBS, Bioman, Taipei City, Taiwan) was used to flush out all spermatozoa from the vas deferens for measurement of sperm parameters.

### 2.2. Biochemical Markers in the Serum

Serum levels of blood urea nitrogen (BUN), creatinine, calcium (Ca), and phosphorus (P) were analyzed by the Union Clinical Laboratory (Taipei City, Taiwan). The ratio of Ca/P was calculated as Ca (mg/dL) divided by P (mg/dL) to define the Ca-P metabolism.

### 2.3. Semen Quality Analysis

Sperm samples obtained as described previously were immediately assessed in terms of sperm function parameters, including sperm motility, sperm count, and morphological abnormality. Samples from all groups were diluted with PBS, and the motility evaluated under a light microscope (E400, Nikon, Tokyo, Japan). Sperm motility was expressed as the percentage of motile sperm over the total spermatozoa counted. At the same time, samples were incubated at 37 °C for 15 min and then diluted with PBS to estimate the sperm count using an automated cell counter (TC20, Bio-Rad, Woodinville, WA, USA) and the percentage of sperm of normal morphology. A drop of sperm sample was placed on a slide and air-dried at room temperature. The dried sperm sample was then fixed with methanol (Honeywell, Redmond, WA, USA) for 5 min and stained with an eosin Y (E4009, Sigma-Aldrich, St. Louis, MO, USA) and ethanol (Bioman) mixture. After 15 min, the slides were rinsed with 75% ethanol (Bioman) and dried. The slides were assessed using a light microscope (DM1000, Leica, Wetzlar, Germany) and the percentage of sperm of normal morphology in a minimum of 100 spermatozoa was assessed. The rest of the sperm samples were centrifuged at 2000× *g* for 6 min. After discarding the supernatant, the sperm pellets were collected and stored at –80 °C.

### 2.4. Testicular Histological Analysis

After tissue processing, formalin-fixed testis tissues were treated at the Department of Pathology of Cardinal Tien Hospital (New Taipei City, Taiwan). Tissues were cut into sections and stained with hematoxylin and eosin (H&E) for pathological examination. Sections of liver and testis tissues were histologically evaluated using light microscopy (DM1000, Leica), and images were captured under 40×, 100×, and 400× magnifications. Testicular seminiferous parameters such as the thickness of the germinal epithelium, mean seminiferous tubule diameter (MSTD), and testicular spermatogenesis were measured. The thickness of the germinal epithelium and diameter of the seminiferous tubule were calculated across the horizontal and vertical axes using ImageJ software (1.50, National Institutes of Health, MD, USA) [24]. The Johnsen score was used to determine testicular spermatogenesis in each group, judging from the level of sperm maturation and scoring from 1 to 10 [25].

### 2.5. Testicular Hormone Levels Analysis

Testis tissues were collected from individual mice and homogenized in RIPA lysis and extraction buffer (Thermo, Chelmsford, MA, USA) containing protease and phosphatase inhibitors (Thermo) and centrifuged at 14,000× *g* for 20 min at 4 °C. The testicular concentration of testosterone was measured using an enzyme-linked immunosorbent assay commercial kit according to the manufacturer’s instructions (Cayman, Item No. 582701, MI, USA).

### 2.6. Western Blot Analysis

Testis tissues were homogenized in RIPA lysis buffer with protease and phosphate inhibitors, then centrifuged at 4 °C for 20 min at 14,000× *g*, and the supernatants were collected. Protein concentrations in each group were determined using a detergent-compatible protein assay (Bio-Rad). Equal quantities of protein (50 μg) were separated by sodium dodecyl sulfate (SDS)-polyacrylamide gels (Bioman) depending on the molecular weight of the desired proteins and then transferred onto polyvinylidene difluoride (PVDF) membranes (GE Healthcare, Freiburg, Germany). Membranes were blocked with 5% (*w*/*v*) nonfat milk at room temperature for 1 h and incubated overnight at 4 °C with primary antibodies. After incubation, the membranes were washed with TBST (diluted from 10× TBST; Omicsbio, Taipei City, Taiwan) and then incubated with secondary antibodies, Goat anti-mouse IgG-HRP (1:5000; sc-2005, Santa Cruz, CA, USA) or Goat anti-rabbit IgG-HRP (1:4000; sc-2054, Santa Cruz), for 1 h at room temperature. Prior to image acquisition, the membranes were incubated in chemiluminescent detection reagent (Omicsbio) for 1 min and signals were observed using a Chemiluminescent Imaging and Analysis System (MiniChemi^TM^ 610, Sage Creation Science, Beijing, China). The protein level of β-actin was used for normalization. The relative expression levels of target proteins were determined using ImageJ software. Primary antibodies including CYP11A1 (1:1000; sc-202456), CYP17A1 (1:1000; sc-66850), 3β-HSD (1:500; sc-28206), 17β-HSD (1:250; sc-135044), StAR (1:1000; sc-25806), Cytochrome C (1:1000; sc-13156), PPARϒ (1:1000; sc-7273), and IL6 (1:1000; sc-57315) were purchased from Santa Cruz. The following primary antibodies were procured from Cell Signaling Technology (Danvers, MA, USA) unless otherwise specified: Bax (1:1000; 2772), Caspase 9 (1:1000; 9508), Caspase 3 (1:500; 9662), Cleaved-Caspase 3 (1:250; 9664), PARP (1:1000; 3542), Caspase 8 (1:1000; 59607, GeneTex, San Antonio, TX, USA), Bcl-xl (1:1000; ab32370, Abcam), TNF-α (1:1000; ab1793, Abcam), NF-κB (1:1000; E381, Abcam), and β-actin (1:10000; A5316, Sigma-Aldrich).

### 2.7. Statistical Analysis

Statistical analyses were conducted using SAS software (9.4, SAS Institute Inc., Cary, NC, USA). Data were expressed as means ± standard deviation (SD). Two-way analysis of variance (ANOVA) with factors of diet and disease followed by Tukey’s post hoc test were used to compare differences in and interactions of mean values between groups. A *p*-value less than 0.05 (*p* < 0.05) was defined as significant.

## 3. Results

### 3.1. Physiological and Serum Biochemical Parameters

**Figure 1:**
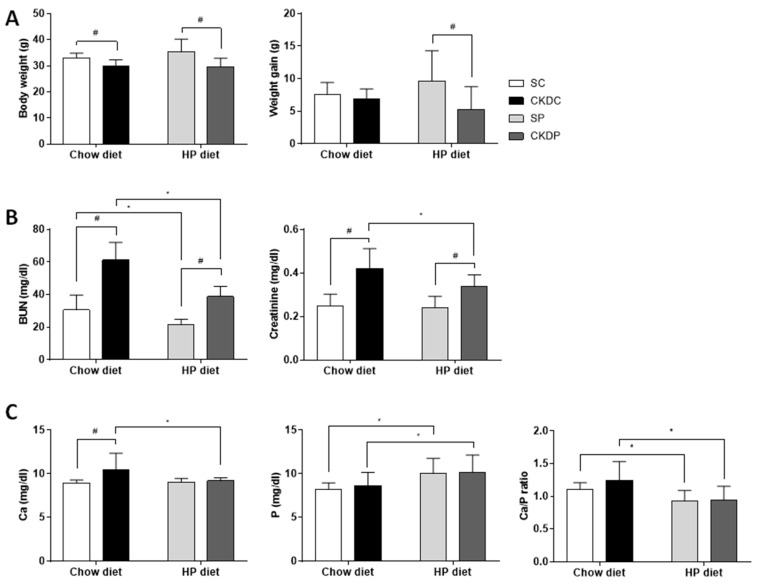
Body weight and blood chemistry levels in high phosphorus diet-fed mice with or without CKD induction. (**A**) Body weight and weight gain, (**B**) renal function, (**C**) calcium, phosphate, and ratio of calcium to phosphate. Data are expressed as the mean ± SD. # indicates a significant difference between the sham-operated and CKD-induced groups (SC vs. CKDC and SP vs. CKDP); * indicates a significant difference between the chow diet and high phosphorus diet groups (SC vs. SP and CKDC vs. CKDP). SC, sham operation group received a chow diet; CKDC, CKD-induced mice received a chow diet; SP, sham operation group received a high phosphorus (HP) diet; CKDP, CKD-induced mice received a HP diet.

After 20 weeks of treatment, the mice with induced chronic kidney disease (CKD) under standard chow and a HP diet showed a significant decrease in body weight as compared with the sham groups (SC and SP), but weight gain throughout this experiment was only higher in the HP-fed sham-operated control mice than in the HP-fed CKD mice (Figure 1A). The CKD groups (CKDC and CKDP) exhibited increases in BUN and creatinine as compared with the sham groups (SC and SP). When compared between diets, the BUN level was significantly lower in the groups fed a HP diet (SP and CKDP), while the creatinine level was only altered in the CKDP group, and no difference was found in the SC or SP groups (Figure 1B). The CKD group with a HP diet displayed a notably higher phosphate level, a lower calcium level and a lower calcium to phosphate ratio as compared with the CKD group fed a chow diet (Figure 1C). In addition, the HP-fed sham-operated control mice had a higher phosphate level than the control mice given a chow diet.

### 3.2. Reproductive Organ Weights and Semen Quality

**Figure 2:**
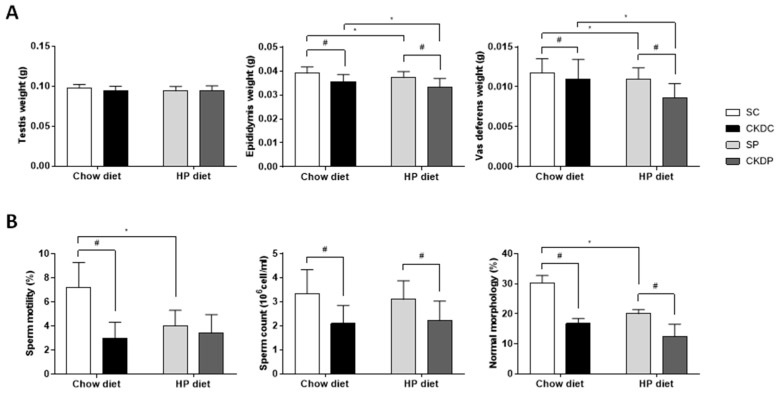
Reproductive organ weights and semen quality in high phosphorus diet-fed mice with or without CKD induction. (**A**) Testis, epididymis, and vas deferens weight, and (**B**) parameters of semen quality. Data are expressed as the mean ± SD. # indicates a significant difference between the sham-operated and CKD-induced groups (SC vs. CKDC and SP vs. CKDP); * indicates a significant difference between the chow diet and high phosphorus diet-fed groups (SC vs. SP and CKDC vs. CKDP).

The testes, epididymis and vas deferens were collected and weighed, and the resulting data are presented in Figure 2A. The induction of CKD caused decreases in the weights of the epididymis and vas deferens in the CKDC and CKDP groups as compared with the SC and SP groups, respectively. Notably, significant effects of the HP diet on the epididymis and vas deferens in the CKD groups were also observed. In terms of semen quality (Figure 2B), the results suggested that a HP diet alone obviously affected sperm motility and the percentage of sperm of normal morphology. The two CKD groups (CKDC and CKDP) exhibited a poorer semen quality, including a lower sperm motility, lower sperm count, and fewer normal forms of sperm than the non-CKD groups; only sperm motility had a lower trend, but there were no significant differences between the SP and CKDP groups. Moreover, an aggravating effect of diet on the reduction in sperm of normal forms was found.

### 3.3. Testicular Morphology and Related Parameters

**Figure 3:**
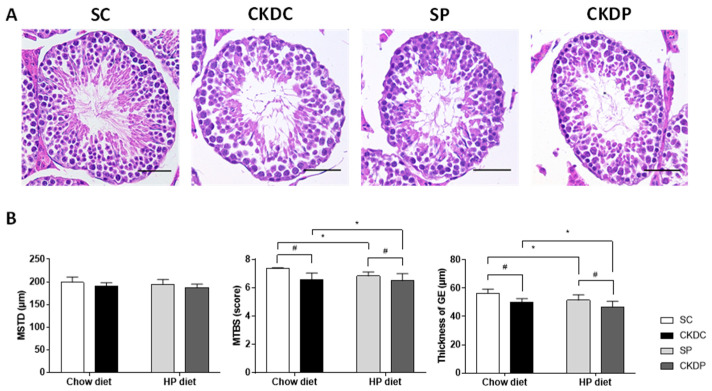
Testicular morphology and related parameters in high phosphorus diet-fed mice with or without CKD induction. (**A**) Testicular morphology, (**B**) mean seminiferous tubule diameter (MSTD), mean testicular biopsy score (MTBS) and thickness of the germinal epithelium (GE). Data are expressed as the mean ± SD. # indicates a significant difference between the sham-operated and CKD-induced groups (SC vs. CKDC and SP vs. CKDP); * indicates a significant difference between the chow diet and high phosphorus diet groups (SC vs. SP and CKDC vs. CKDP).

Testes sections stained by hematoxylin and eosin (H&E) are shown in Figure 3A. The sham group had a normal histological structure with complete spermatogenesis. Marked histological alterations were found in the HP-fed and CKD-induced groups (SP, CKDC, and CKDP), with scattered cell divisions during spermatogenesis and decreasing numbers of elongated spermatids and spermatozoa being observed. Parameters such as the diameter of seminiferous tubules, germinal epithelium thickness and scoring of spermatogenesis were calculated (Figure 3B). Significant reductions in the germinal epithelium thickness and the biopsy score were observed in the CKD-induced and HP groups, with the exception of the difference in biopsy score between the SP and CKDP groups. No obvious differences were observed in the mean length of the testicular seminiferous tubules between the four groups.

### 3.4. Testicular Testosterone Level and Protein Expressions of Testosterone Biosynthesis Enzymes in the Testis

**Figure 4:**
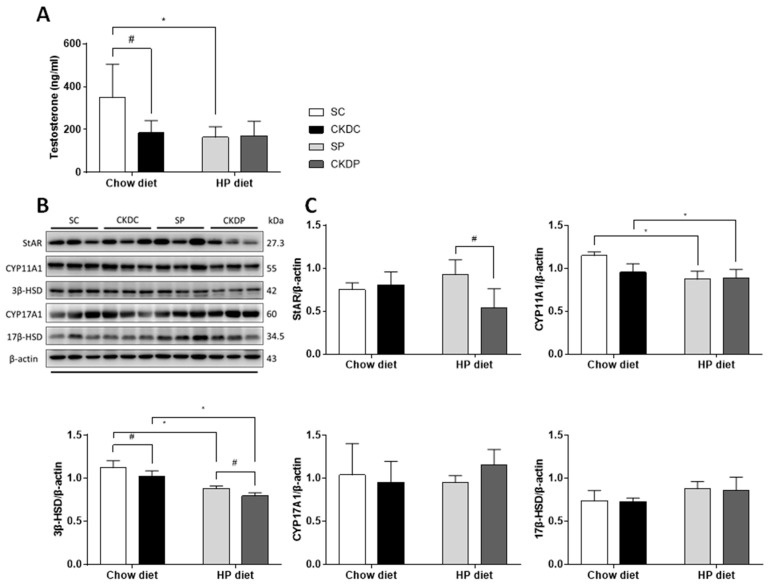
Testicular testosterone levels and protein expressions of testosterone biosynthesis markers in high phosphorus diet-fed mice with or without CKD induction. (**A**) Testicular testosterone levels; (**B**) testosterone biosynthesis enzymes (StAR, CYP11A1, 3β-HSD, CYP17A1, and 17 β-HSD) and (**C**) relative density analysis of the protein bands. Data are expressed as the mean ± SD. # indicates a significant difference between the sham-operated and CKD-induced groups (SC vs. CKDC and SP vs. CKDP); * indicates a significant difference between the chow diet and high phosphorus diet groups (SC vs. SP and CKDC vs. CKDP).

As testosterone takes part in maintaining sperm production and function, the testicular testosterone level was therefore measured. The testosterone level was notably lower in the CKD-induced and HP diet only groups (CKDC and SP) than in the control group (SC). Furthermore, the protein expressions of enzymes related to testosterone biosynthesis were examined to determine whether the HP diet and CKD affected these regulators or not. The results showed that the mice with induced CKD exhibited statistically significant downregulations of CYP11A1 and 3β-HSD proteins in the testis. In the case of the HP-fed control mice, similarly reduced expressions of CYP11A1 and 3β-HSD proteins were found as compared with the sham group (Figure 4). A high phosphate intake in the CKD mice had no effect on testicular testosterone levels or protein expressions of enzymes involved in testosterone production.

### 3.5. Oxidative Stress Biomarkers in Testis

**Figure 5:**
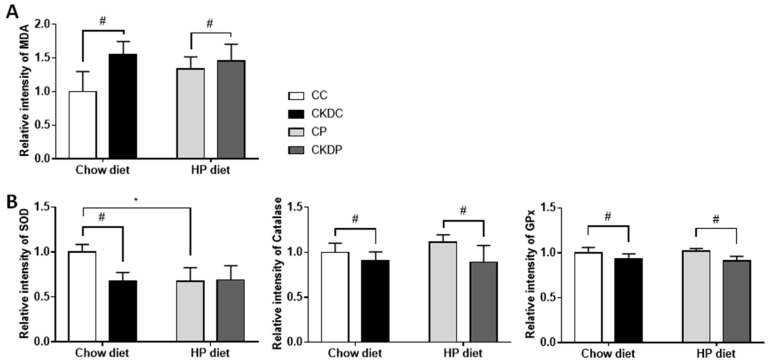
Testicular oxidative stress biomarkers in high phosphorus diet-fed mice with or without CKD induction. (**A**) MDA level and (**B**) antioxidant activities (SOD, CAT and GPx). Data are expressed as the mean ± SD. # indicates a significant difference between the sham-operated and CKD-induced groups (SC vs. CKDC and SP vs. CKDP); * indicates a significant difference between the chow diet and high phosphorus diet groups (SC vs. SP and CKDC vs. CKDP). SOD, superoxide dismutase; CAT, catalase; GPx, glutathione peroxidase; MDA, malondialdehyde.

Analysis of testicular antioxidants revealed markedly decreased activities of SOD and GPx in the CKDC group, and Catalase and GPx in the CKDP group, as compared with the sham groups (SC and SP). Contrary to the sham-operated mice fed the standard chow diet, the SOD activity was much lower in the mice fed a HP diet. Moreover, an increased content of MDA in the CKD-induced group was observed, indicating an adverse effect of CKD on the testicular antioxidative system (Figure 5).

### 3.6. Protein Expressions of Apoptosis Markers in the Testis

**Figure 6:**
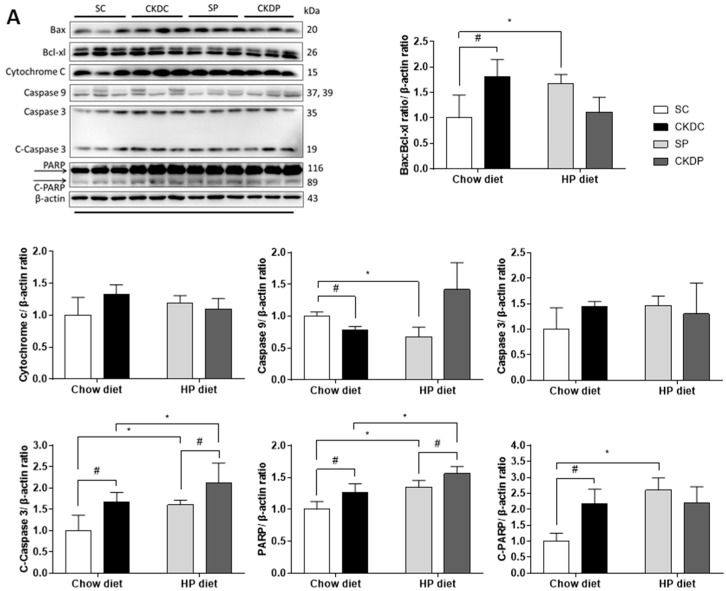

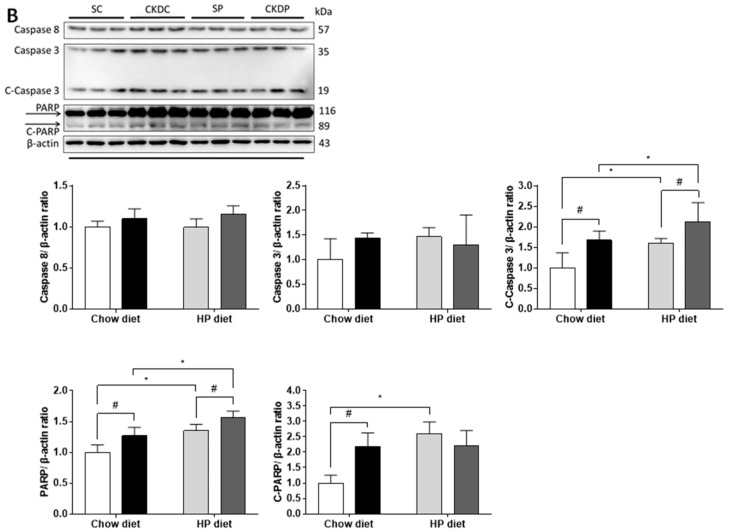
Protein expressions of testicular apoptosis pathway regulators in high phosphorus diet-fed mice with or without CKD induction, including (**A**) extrinsic and (**B**) intrinsic pathway markers. Data are expressed as the mean ± SD. # indicates a significant difference between the sham-operated and CKD-induced groups (SC vs. CKDC and SP vs. CKDP); * indicates a significant difference between the chow diet and HP diet groups (SC vs. SP and CKDC vs. CKDP).

In an attempt to identify possible mechanisms related to CKD and HP diet-induced testicular dysfunction, apoptosis regulators were analyzed by Western blot. Extrinsic apoptosis regulators, including the ratio of Bax and Bcl-xl, intrinsic regulator caspase 9, and downstream effectors including PARP and the cleaved forms of caspase 3 and PARP in the testes were upregulated under CKD induction. In the non-CKD groups, the HP diet resulted in significant elevations in the ratio of Bax and Bcl-xl, PARP, and cleaved forms of caspase 3 and PARP as compared with the CKD-induced groups. Phosphate intervention induced high levels of caspase 9 and PARP but decreased the ratio of Bax and Bcl-xl (Figure 6).

### 3.7. Protein Expressions of Inflammation Markers in the Testis

**Figure 7:**
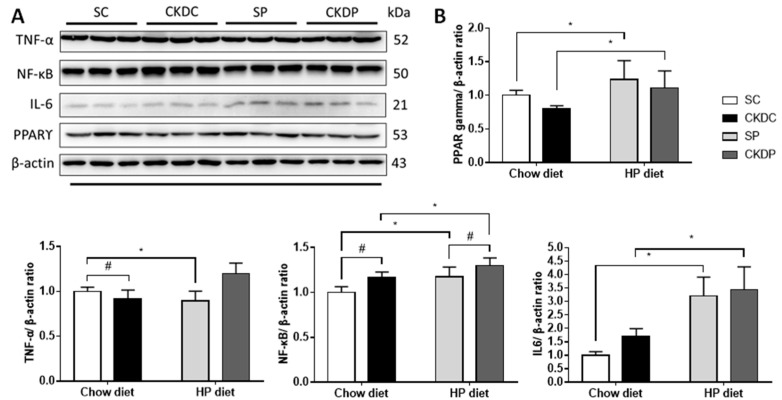
Protein expressions of testicular inflammation pathway markers in high phosphorus diet-fed mice with or without CKD induction. (**A**) Protein expressions of inflammation pathway markers (TNF-α, NF-κB, IL-6 and PPARγ) and β-actin was used for normalization; (**B**) relative density analysis of the protein bands. Data are expressed as the mean ± SD. # indicates a significant difference between the sham-operated and CKD-induced groups (SC vs. CKDC and SP vs. CKDP); * indicates a significant difference between the chow diet and high phosphorus diet groups (SC vs. SP and CKDC vs. CKDP).

Examination of inflammation-related proteins showed increased TNF-α and NF-κB expressions in the CKD groups. With higher levels of phosphate supplementation in the CKD mice, notable increases in the TNF-αand IL-6 protein expressions were observed as compared with the chow diet groups. Furthermore, statistically significant differences were observed in the protein expression levels of NF-κB and IL-6 in the SC and SP groups. It appeared that the anti-inflammatory effect of PPAR-ϒ was similar in each group (Figure 7).

## 4. Discussion

In this study, a murine 5/6 nephrectomy model was established to induce CKD, which is the most frequently-used model in studies of progressive renal failure [26,27]. The effect of 5/6 nephrectomy was confirmed by examination of the functions of the remnant kidney, assessed from biochemical values and histologic analysis [28], and significant elevations in the blood BUN and creatinine levels were observed in the CKD-induced groups, in agreement with the results of other studies that employed the same model [29,30]. A high serum phosphate concentration is a common complication in the progress of CKD. Both CKD and hyperphosphatemia have adverse effects on fertility [12,13]. Therefore, this study hypothesized that phosphate would aggregate CKD-induced negative reproductive outcomes, in turn confirming the importance of phosphate restriction in CKD to achieve a better male fertility and life quality. In addition, this study also included a group of normal mice fed a HP diet to represent people who consume excessive phosphorus, as dietary phosphate toxicity has been recognized as an emerging problem that may be caused by high intakes of processed foods and carbonated beverages [31].

In the present study, the groups on a HP diet manifested higher serum phosphate levels, indicating excess phosphate loading. Phosphate loading increased the BUN level in the control mice; however, in contrast, improved renal function but more severe disruption in mineral metabolism were observed in the CKD mice. Other studies have shown similar improvement in kidney function in a 5/6 nephrectomy model [32,33]. However, another study observed kidney dysfunction associated with a HP diet [34]. Phosphate might have an interactive effect with surgery-induced renal damage, and the related mechanisms remain to be clarified.

Maturation and motility development of sperm take place in the epididymis, and then mature sperm are stored in the vas deferens and transported to the ejaculatory duct during ejaculation [35]. Reduced reproductive organ weights could be a consequence of impaired sperm function, in accordance with the observation of a lower semen quality. In the testicular morphology, CKD exhibited worsening spermatogenesis, characterized by a decreasing epithelium thickness and poor sperm production, which may lead to a low (oligospermia) or zero (azoospermia) sperm count [36,37]. Testosterone and its synthesis enzymes, CYP11A1 and 3β-HSD, were also decreased. CYP11A1 catalyzes the production of pregnenolone from cholesterol, while 3β-HSD is a critical enzyme involved in the conversion of pregnenolone to dihydrotestosterone and a marker highly-expressed in Leydig cells [38,39]. A low testosterone concentration is also considered a key effector of erectile dysfunction [40]. CKD+HP resulted in aggravation of reduced epididymis and vas deferens weights accompanied by higher sperm morphology abnormality and a lower expression of StAR, a rate-limiting enzyme in testosterone biosynthesis [41]. Concerning the healthy mice, a HP diet induced damage to the testicular morphology and resulted in a lower semen quality as well as lower testosterone levels, along with reduced CYP11A1 and 3β-HSD expressions. Phosphorus excess has been demonstrated to speed up the aging process, and premature aging affects organ function, which may inflict infertility [42,43].

The commonly-studied mechanisms of infertility involve oxidative stress, apoptosis, and inflammation, and these effectors interact with each other [44,45]. Further experiments revealed that CKD suppressed the antioxidative defense system and induced MDA overproduction. Apoptosis mediators and inflammatory cytokines were also upregulated. An impaired antioxidant system in CKD has been reported in human studies and animal models [46,47,48]. Downregulation of SOD contributes to an elevation in superoxide, which can further stimulate NF-κB activation and induce inflammation in CKD [49,50,51]. Moreover, the uremic toxins produced in the progression of CKD may initiate inflammation and activate cytokines and radical species and thus increase oxidative stress [52]. Elevated inflammation has been shown to be correlated with testosterone deficiency [53]. In terms of apoptosis, apoptosis has a critical role in the removal of abnormal cells in spermatogenesis [54]. The results of the present study also showed damaged stages of spermatogenesis and sperm morphology accompanied by higher expressions of apoptosis mediators, which help to remove dysfunctional spermatocytes. Moreover, NF-κB-dependent inflammation is capable of regulating apoptosis [55]. The CKD+HP group exhibited similar changes in markers of oxidative stress and apoptosis but higher expressions of inflammatory cytokines, indicating worsened inflammation in the testis. Yamada et al. pointed out the direct relationship of dietary phosphate and inflammation, leading to increased malnutrition and vascular calcification [21]. Martínez-Moreno et al. found that high phosphate loading induced pro-inflammatory and pro-oxidative reactions [56]. On the other hand, a HP diet not only decreased the activity of SOD but also induced apoptosis and inflammation in the normal mice, which helps to make sense of previous data related to testis damage and sperm dysfunction.

## 5. Conclusions

In conclusion, the results of our study showed significant adverse effects of CKD and related mechanisms on male infertility. Supplementation of phosphate may aggravate the damage to the testis and sperm function through inflammation. The finding of excess dietary phosphorus in healthy subjects suggests that this risk factor that decreases male reproductive function is being ignored.

## Figures and Tables

**Table 1:** Nutritional composition of the chow diet and the high phosphorus (HP) diet.

Nutrient (%)	Chow	HP
Protein	24.6	18.2
Fat (ether extract)	5.0	7.1
Fat (acid hydrolysis)	6.4	7.1
Fiber	4.2	5.0
Nitrogen-free extract	50.0	58.5
Starch	29.4	43.0
Sucrose	1.2	12.4
Calcium	1.0	0.5
Phosphorus	0.8	2.0
**Energy (%)**	**Chow**	**HP**
Carbohydrate	58	63
Fat	13	17
Protein	29	20

Chow diet: LabDiet 5010; HP diet: TestDiet AIN-93G w/2% phosphorus/peanut butter flavored.
